# Increased inflammation is associated with islet autoimmunity and type 1 diabetes in the Diabetes Autoimmunity Study in the Young (DAISY)

**DOI:** 10.1371/journal.pone.0174840

**Published:** 2017-04-05

**Authors:** Kathleen Waugh, Janet Snell-Bergeon, Aaron Michels, Fran Dong, Andrea K. Steck, Brigitte I. Frohnert, Jill M. Norris, Marian Rewers

**Affiliations:** 1 Barbara Davis Center for Diabetes, University of Colorado Denver, Aurora, Colorado, United States of America; 2 Department of Epidemiology, Colorado School of Public Health, University of Colorado, Aurora, Colorado, United States of America; Weizmann Institute of Science, ISRAEL

## Abstract

**Background:**

Type 1 diabetes (TID) is characterized by a loss of pancreatic islet beta cell function resulting in loss of insulin production. Genetic and environmental factors may trigger immune responses targeting beta cells thus generating islet antibodies (IA). Immune response pathways involve a cascade of events, initiated by cytokines and chemokines, producing inflammation which can result in tissue damage.

**Methods:**

A nested case-control study was performed to identify temporal changes in cytokine levels in 75 DAISY subjects: 25 diagnosed T1D, 25 persistent IA, and 25 controls. Serum samples were selected at four time points: (T1) earliest, (T2) just prior to IA, (T3) just after IA, and (T4) prior to T1D diagnosis or most recent. Cytokines (IFN-α2a, IL-6, IL-17, IL-1β, IP-10, MCP-1, IFN-γ, IL-1α, and IL-1ra) were measured using the Meso Scale Discovery system Human Custom Cytokine 9-Plex assay.

**Results:**

Multivariate mixed models adjusting for HLA risk, first-degree relative status, age, and gender, showed MCP-1 and IFN-үto be significantly higher at T3 in T1D compared to IA subjects. At T4, IP-10 was significantly higher in IA subjects than controls.

**Conclusions:**

This repeated measures nested case-control study identified increased inflammatory markers in IA children who developed T1D compared to IA children who had not progressed to clinical disease. It also showed increased inflammation in both T1D and IA children when compared to controls. Results suggest inflammation may be related to both the development of IA and progression to T1D.

## Introduction

Type 1 diabetes (T1D) affects approximately 1.5 million people in the United States, with the incidence increasing over the past several decades worldwide. The complications of type 1 diabetes lead to an increased healthcare burden and costs estimated to be more than $7,000 per person annually[1, 2]. While type 1 diabetes has a strong genetic component, the increasing incidence must be attributable to environmental triggers. In recent years, an area of investigation has focused on how the innate immune system may be involved in the pathogenesis of T1D. Insults, such as microbial infections, initiate the innate immune system response and a cascade of events, including the expression of pro-inflammatory cytokines and chemokines occurs. These findings raise the question does systemic inflammation exist in the context of islet autoimmunity and T1D? It is well appreciated that type 2 diabetes has systemic inflammation as a prominent factor in disease pathogenesis[3]; however results in autoimmune diabetes, including latent autoimmune diabetes of adulthood and T1D, is less clear[4]. A longitudinal study measuring the inflammatory marker C-reactive protein (CRP) in islet autoantibody subjects over time that progress to T1D, indicated that CRP concentrations are not a valuable marker of progression to T1D[5]. In new-onset T1D subjects, compared to healthy controls, a differential expression in sera of some cytokines and chemokines has been observed [6, 7]. Also, inflammation of the pancreatic islet cells and increased inflammatory markers have been reported in children with T1D, particularly at diagnosis [8, 9]. Further, we have seen a positive association between enterovirus infection, detected in serum, and progression from islet autoimmunity (IA) to T1D [10]. Therefore, it is hypothesized that the activation of cytokines and resulting inflammation may play a role in the development of IA and subsequent progression to T1D.

The Diabetes Autoimmunity Study in the Young (DAISY) is following children with genetic or familial risk for type 1 diabetes in order to determine which environmental factors influence the risk for developing IA and clinical T1D[11]. The aim of this study was to examine whether inflammatory cytokines and chemokines are increased prior to the development of either IA or T1D.

## Materials and methods

### Study population

To identify potential circulating serum cytokines associated with development of IA and T1D, we performed a nested case-control study of children participating in the DAISY study. DAISY is a prospective cohort of children at increased risk for type 1 diabetes. Participants are either T1D first-degree relatives (N = 1,123) or general population children who carry type 1 diabetes-susceptibility HLA-DR, DQ genotypes (N = 1,424). The details of the newborn screening and follow-up have been previously published [11, 12]. Recruitment took place between 1993 and 2004 and follow-up results through July of 2011 were included in this analysis. Written informed consent was obtained from the parents of study participants. The Colorado Multiple Institutional Review Board approved all study protocols.

### Study endpoints

Study subjects were considered persistently IA positive if they had at least two consecutive, confirmed islet autoantibody positive samples, not due to maternal autoantibody transfer, or if they had one confirmed autoantibody positive sample and developed T1D prior to the next sample collection. Autoantibodies were tested at 9, 15, and 24 months of age and, if negative, annually thereafter; children found to be autoantibody positive were re-tested every 3–6 months. Radio-immunoassays were used to measure serum autoantibodies to insulin (IAA), GAD_65_ (GAA), and IA-2 (BDC512), as previously described [13–15], with rigorous duplicate testing and confirmation of all positive and a subset of negative results. All samples from children ever positive for any of the above autoantibodies and from those who developed T1D were retrospectively tested for autoantibodies to zinc transporter 8 (ZnT8A) [16]. The last sample from autoantibody negative active study participants was also tested for ZnT8A. The cut-off for positivity was established as the 99th percentile of healthy controls. The inter-assay coefficients of variation for IAA, GAA, IA-2A, and ZnT8A were 20%, 10%, 5%, and 10.4%, respectively. In the 2010 Diabetes Autoantibody Standardization Program (DASP) workshop, the sensitivity and specificity for IAA were 56% and 99%, respectively, 82% and 99% for GADA, 66% and 99% for IA-2A, and 64% and 100% for ZnT8A[17]. Type 1 diabetes was diagnosed by a physician using the American Diabetes Association criteria.

### Selection of subjects for cytokine studies

Seventy-five children followed prospectively for development of IA and T1D were selected from the DAISY cohort. Of those, 25 children were followed to type 1 diabetes (**T1D group**), 25 were persistently positive for at least one islet autoantibody and not diagnosed with T1D at their last study visit (**IA group**), and 25 controls who were previously negative for all islet autoantibodies at all DAISY visits (**C group**). Controls were frequency matched to the combined T1D and IA group subjects based upon age, HLA DR/DQ genotypes, gender and family history of T1D.

### Sample selection

Serum samples were selected primarily with regard to T1D follow-up time. Controls were frequency matched on 1) the T1D and IA groups combined HLA genotypes, gender, and family history of T1D and 2) the age of T1D’s at each time point, defined below:

**T1** is the earliest sample available, for all groups. Participants for all groups were enrolled by 9 months of age, but a serum sample was not necessarily available from each first visit so the earliest available sample was selected.

**T2** is selected based on the seroconversion visit of the T1D and IA participants. The closest available sample prior to seroconversion was selected.

**T3** is also selected based on the seroconversion visit of the T1D and IA participants. The closest available sample just after seroconversion was selected.

**T4** is the last sample available. For T1D participants, this would be the last sample available prior to diagnosis. For IA participants, this would be the last sample available at the time of the assay.

At each clinic visit, blood was drawn into a serum separator tube, allowed to clot for 15 minutes and spun for 10 minutes to isolate serum. Serum was aliquoted into multiple 1.5ml cryovials and immediately stored at -80°C.

### Assay specifications and procedures

Serum samples from clinic visits, thawed on ice, were analyzed using a customized Meso Scale Discovery^TM^ immunoassay, MSD Human Custom Cytokine 9-Plex US. This technology includes biotinylated and conjugated SULFO-TAG detection antibodies to measure nine different analytes in one microtiter well. Pristine serum from each subject at each time point (50ul) was thawed and analyzed in duplicate for nine cytokines per manufacturer recommendations: interferon (IFN)-α2a, interleukin (IL)-6, IL-17, IL-1β, interferon gamma-induced protein (IP)-10, monocyte chemotactic protein (MCP)-1, IFN-γ, IL-1α, and IL-1ra (IL-1 receptor antagonist).

### Statistical analysis

We used two-sample t-tests and ANOVA tests for univariate comparison of continuous variables. Chi-square tests and Fisher exact tests were used for statistical comparisons for dichotomous and categorical variables. Values of p≤ 0.05 were considered to be statistically significant. Generalized linear mixed models were used to examine the cytokine levels as repeated measures over time in the study groups. For this analysis, Proc Mixed in SAS version 9.4 (SAS Institute, Cary, NC) was used. Potential confounding due to differences in age, sex, and FDR status was addressed in our regression analysis. In addition, the mixed models were adjusted for gender and enrollment cohort (general population cohort versus first-degree relative (FDR)). Estimates were obtained from the mixed models using estimate statements in a cell means model.

Samples were unavailable at T1 for three T1D subjects and at T2 for six T1D subjects. Five of the nine cytokines (IL-1β, IL-1ra, IL-1α, IL-17, IFN-α2a) had more than 50% of the values below the lower limit of detection (LLD). These cytokines were excluded from the analysis (S1 Table). For the remaining cytokines (IFN-ү, IL-6, IP-10, and MCP-1), all values under the LLD were censored at the LLD for analysis.

Log ten transformation was performed for the cytokine data in order to normalize the distribution.

## Results

Characteristics of DAISY nested case-control study subjects are shown in Table 1. There were no significant differences in sex, ethnicity or HLA-DR genotypes. Although frequency matching was attempted, there remained a significant difference in age, only at T2 and T3, while ages are similar in all groups at T1 and T4. The BMI z-score, evaluated at each time point, was not significantly different across groups (S1 Fig).

**Table 1:** Characteristics of DAISY nested case-control study.

		T1D	IA	Control	p-value
Number of subjects		25	25	25	
Gender (male)		15	15	12	0.61
T1D first degree relative		17	14	10	0.07
Race/ethnicity					0.051
	Non-Hispanic White	24	17	20	
	Hispanic	1	6	5	
	Other	0	2	0	
HLA					0.375
	DR 3/4	11	7	4	
	DR 3/3 or 3/X	5	3	5	
	DR 4/4 or 4/X	7	11	13	
	DR X/X	2	4	3	
Age (mean, yrs)					
	T1	1.4	1.6	0.9	0.058
	T2	4.6	6.2	3.6	0.008
	T3	6.2	8.8	6.4	0.007
	T4	10.5	12.6	10.9	0.13

The univariate log means for all cytokines by visit and study group (S2 Table) demonstrated significant differences at T3, just after the development of IA, for monocyte chemotactic protein 1 (MCP-1) and. The log mean concentration of MCP-1 was significantly higher in subjects that progressed to T1D when compared to IA subjects (p = 0.006) but not different from controls.

A multivariate analysis, adjusted for age, FDR and sex was conducted to determine differences in cytokine concentrations among the groups. As shown in Table 2, MCP-1, also referred to as CCL2, was significantly lower at T3, in IA subjects when compared to controls (p = 0.02) and at T2 and T3 when compared to T1D subjects (p = 0.03, 0.02 respectively). IP-10, also known as CXCL10 was significantly higher in IA subjects than controls at T4 (p = 0.034).

**Table 2:** Difference in Log Means Concentration of Cytokines (Adjusted for gender, age, HLA and FDR status).

	T1D vs. Control	IA vs. Control	IA vs. T1D	Overall p-value
**IL-6**				0.75
T1	0.016	-0.119	-0.135	
T2	0.166	0.216	0.050	
T3	0.080	0.191	0.111	
T4	0.140	-0.186	-0.326	
**IP-10**				0.27
T1	-0.003	0.028	0.031	
T2	0.025	0.094	0.070	
T3	0.077	0.173	0.096	
T4	0.112	0.407*	0.295	
**MCP-1**				0.008
T1	-0.102	-0.172	-0.07	
T2	0.071	-0.159	-0.229*	
T3	0.052	-0.194*	-0.246*	
T4	0.008	-0.170	-0.178	
**IFN-γ**				0.08
T1	-0.244	0.027	0.271	
T2	-0.298	-0.531	-0.233	
T3	0.355	-0.298	-0.653	
T4	0.461	0.651	0.190	

*p<0.05

**Fig 1:**
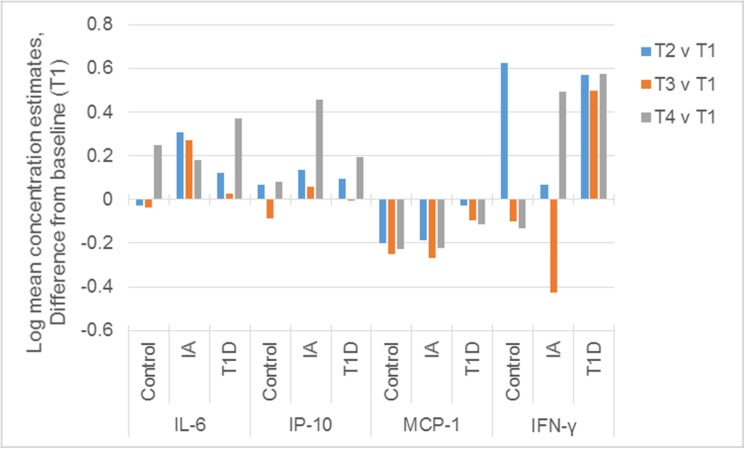
The Mixed Model Estimate of Change from Time 1. Pattern of change in serum cytokine or chemokine concentrations over time for the T1D, IA, and control groups. The log mean concentration for each individual cytokine is depicted as the difference from T1, the baseline (T2-T1, T3-T1, T4-T1).

We next examined the pattern of change in serum cytokine concentrations over time for each group, presented in Fig 1. While IFN-γ increased between T1 and T2 in both the cohort progressing to T1D and the controls, this increase persisted in only the T1D cohort, which was elevated at all time points after T1. IL-6 was somewhat increased over baseline at T2 and T3 in the IA and T1D groups but by T4, IL-6 was elevated in all groups. Interestingly, IL-6 was elevated at all time points after T1 in the IA group. The pattern of change over time for IP-10 and MCP-1 did not dramatically differ between the groups.

## Discussion

This serial sample analysis for serum cytokines showed significant differences in cytokine concentrations between participants who progressed to T1D, those who had IA but did not progress to T1D, and controls without IA, at the time of seroconversion, T3, and just prior to diagnosis, T4. In addition, among the three comparison groups, different patterns of change occurred in some serum cytokine concentrations from the earliest time point to the last time point. These results suggest early alterations in immune response pathways.

This analysis did not demonstrate profoundly distinct cytokine profiles for our IA or T1D group compared to controls; however, it did show significant differences between these groups at specific stages of autoimmunity progression, including between those with IA who did and did not progress to T1D. This customized assay also corresponded to results previously demonstrated from ELISA assays[18] and peripheral blood mononuclear cell (PBMC) stimulation assays[6, 19–21]. The concentration of IP-10 was greater at T4 in the IA group compared to controls. IP-10, also referred to as CXCL10, is characterized as an inflammatory cytokine that results in recruitment of lymphoctyes, especially T cells and NK T cells to sites of inflammation. There is conflicting data regarding IP-10 levels in IA and T1D, with some previous studies indicating no difference between IA and controls[18] while others indicate lower levels in IA compared to controls[21]. Our results showing higher serum IP-10 concentrations in those at risk agree with several previous reports[22, 23]Importantly, this analysis is the first to examine serial samples through the progression of autoimmunity as opposed to a single timepoint in the disease course and may represent a more complete picture of serum cytokine levels.

We observed elevated levels of other pro-inflammatory cytokines, IFN-γ and IL-6 IFN-γ is elevated after seroconversion in both IA and T1D groups, although delayed in the IA group, implicating inflammatory mediators just prior to T1D diagnosis. This is consistent with other reports of IFN-γ involvement in beta cell damage and apoptosis[24, 25]. IL-6 has been shown to participate in the balance of pro-inflammatoryTh17 and suppressor lymphocytes,Treg cells, during an immune response and others report elevated levels of IL-6 in blood serum of T1D patients[26]. Our results indicate a moderate yet persistent increase in IL-6 in the cohort that progressed to T1D compared to controls, across all time points.

We observed significantly lower levels of MCP-1 in IA compared to both controls and the T1D group around the time of seroconversion, T2 and T3. However, T1D subjects had similar levels to controls at all time points. MCP-1 is capable of being induced from PBMCs of newly diagnosed T1D children when stimulated with GAD_65_ but is not significantly elevated without an in vitro stimulus[20], which is one possibility why we did not see a difference between these groups. However, the IA individuals had lower MCP-1 levels than controls and T1D patientsThese data raise the possiblity that basal serum levels of MCP-1 may not be reflective of pathology in target tissues or an in vitro stimulus is needed to define differences based upon stage of the disease process[21].

The longitudinal nature of the DAISY cohort has allowed us to analyze changes in circulating cytokine levels at multiple time points along the progression to autoimmunity and development of clinical T1D. While we attempted to determine patterns and trends over four time points, we were still limited to four snapshots in time over the course of several years of autoimmunity progression. We also utilized a customized assay plate which allowed us to measure multiple analytes in a single well from one sample, therefore enabling us to analyze multiple cytokines among several samples relatively quickly. The limitations to our approach include the fact that predominantly inflammatory cytokines and chemokines were measured and that our sample size is relatively small. Future studies will analyze both inflammatory and regulatory cytokines, which have been reported from PBMCs stimulated with islet peptides in both T1D and healthly controls[27, 28]. Another limitation was that ages were not matched at all timepoints, as the mean age of the IA positive group remained significantly different from the T1D group at the time of seroconversion. It has previously been reported, in this cohort and others, that progression to T1D is associated with age of islet autoantibody appearance[29–31] and as we see in this analysis, those that remain IA positive for an extended period of follow up develop autoimmunity at a later age than those that progress to T1D more quickly. However, despite the age difference, the mean time of islet autoimmunity (seroconversion to T4) in the IA positive and T1D groups are similar, 4.4 +/- 3.2 and 5.3 +/- 2.3 years (p = 0.278), respectively. Furthermore, we adjusted for age in the multivariate analysis.

In conclusion, the results of this study suggest that inflammation may be related to the development and progression to T1D, and there may be multiple levels of altered immune regulation, demonstrated by the differences between the IA and T1D groups. Future studies focusing on identifying the differences between these two groups in terms of inflammatory and regulatory cytokines may define optimal timing for interventions to delay or prevent progression to islet autoimmunity and clinical T1D.

## Supporting information

S1 Fig Distribution of BMI z-score at each time point. The BMI z-score distribution for each group at each time point. At time point 1 (T1) n = 11. Many participants are less than 1 year of age at T1 and length was not measured, therefore BMI and BMI z-score could not be calculated-no controls had recorded length measurements at T1.
(TIF)

S1 Table Percent censored and lower limit of detection (LLD) for each cytokine. Cytokine output in which greater that 50% of the data was censored was excluded from the analysis.
(DOCX)

S2 Table Log mean concentration of cytokine by group and time (unadjusted). Univariate analysis of the log mean concentration for cytokines included in the analysis, by group and time point. *P-value of ≤ 0.05.
(DOCX)

S1 Dataset Original data used for the analyses included in this manuscript. (XLSX)

## References

[1] Lee JM, Sundaram V, Sanders L, Chamberlain L, Wise P. Health Care Utilization and Costs of Publicly-Insured Children with Diabetes in California. J Pediatr. 2015;167(2):449–54 e6. 10.1016/j.jpeds.2015.04.067 26028286

[2] Shrestha SS, Zhang P, Albright A, Imperatore G. Medical expenditures associated with diabetes among privately insured U.S. youth in 2007. Diabetes Care. 2011;34(5):1097–101. 10.2337/dc10-2177 21525502 PMC3114503

[3] Pietropaolo M, Barinas-Mitchell E, Kuller LH. The heterogeneity of diabetes: unraveling a dispute: is systemic inflammation related to islet autoimmunity? Diabetes. 2007;56(5):1189–97. 10.2337/db06-0880 17322478

[4] Hawa MI, Thivolet C, Mauricio D, Alemanno I, Cipponeri E, Collier D, et al Metabolic syndrome and autoimmune diabetes: action LADA 3. Diabetes Care. 2009;32(1):160–4. 10.2337/dc08-1419 18945926 PMC2606853

[5] Bonifacio E, Mollenhauer U, Buuck D, Ziegler AG. C-reactive protein concentration is not related to islet autoimmunity status in offspring of parents with type 1 diabetes. Clin Immunol. 2005;115(2):173–7. 10.1016/j.clim.2005.01.004 15885640

[6] Meyers AJ, Shah RR, Gottlieb PA, Zipris D. Altered Toll-like receptor signaling pathways in human type 1 diabetes. J Mol Med (Berl). 2010;88(12):1221–31. 20725710 10.1007/s00109-010-0666-6

[7] Cabrera SM, Henschel AM, Hessner MJ. Innate inflammation in type 1 diabetes. Transl Res. 2016;167(1):214–27. 10.1016/j.trsl.2015.04.011 25980926 PMC4626442

[8] Dogan Y, Akarsu S, Ustundag B, Yilmaz E, Gurgoze MK. Serum IL-1beta, IL-2, and IL-6 in insulin-dependent diabetic children. Mediators Inflamm. 2006;2006(1):59206 10.1155/MI/2006/59206 16864906 PMC1570393

[9] Chatzigeorgiou A, Harokopos V, Mylona-Karagianni C, Tsouvalas E, Aidinis V, Kamper EF. The pattern of inflammatory/anti-inflammatory cytokines and chemokines in type 1 diabetic patients over time. Ann Med. 2010;42(6):426–38. 10.3109/07853890.2010.495951 20568978

[10] Stene LC, Oikarinen S, Hyoty H, Barriga KJ, Norris JM, Klingensmith G, et al Enterovirus infection and progression from islet autoimmunity to type 1 diabetes: the Diabetes and Autoimmunity Study in the Young (DAISY). Diabetes. 2010;59(12):3174–80. 10.2337/db10-0866 20858685 PMC2992780

[11] Rewers M, Bugawan TL, Norris JM, Blair A, Beaty B, Hoffman M, et al Newborn screening for HLA markers associated with IDDM: diabetes autoimmunity study in the young (DAISY). Diabetologia. 1996;39(7):807–12. 8817105 10.1007/s001250050514

[12] Rewers M, Norris JM, Eisenbarth GS, Erlich HA, Beaty B, Klingensmith G, et al Beta-cell autoantibodies in infants and toddlers without IDDM relatives: diabetes autoimmunity study in the young (DAISY). J Autoimmun. 1996;9(3):405–10. 8816978 10.1006/jaut.1996.0055

[13] Vardi P, Dib SA, Tuttleman M, Connelly JE, Grinbergs M, Radizabeh A, et al Competitive insulin autoantibody assay. Prospective evaluation of subjects at high risk for development of type I diabetes mellitus. Diabetes. 1987;36(11):1286–91. 3666319 10.2337/diab.36.11.1286

[14] Grubin CE, Daniels T, Toivola B, Landin-Olsson M, Hagopian WA, Li L, et al A novel radioligand binding assay to determine diagnostic accuracy of isoform-specific glutamic acid decarboxylase antibodies in childhood IDDM. Diabetologia. 1994;37(4):344–50. 8063033 10.1007/BF00408469

[15] Gianani R, Rabin DU, Verge CF, Yu L, Babu SR, Pietropaolo M, et al ICA512 autoantibody radioassay. Diabetes. 1995;44(11):1340–4. 7589834 10.2337/diab.44.11.1340

[16] Yu L, Boulware DC, Beam CA, Hutton JC, Wenzlau JM, Greenbaum CJ, et al Zinc transporter-8 autoantibodies improve prediction of type 1 diabetes in relatives positive for the standard biochemical autoantibodies. Diabetes Care. 2012;35(6):1213–8. 10.2337/dc11-2081 22446173 PMC3357246

[17] Bingley PJ, Bonifacio E, Mueller PW. Diabetes Antibody Standardization Program: first assay proficiency evaluation. Diabetes. 2003;52(5):1128–36. 12716742 10.2337/diabetes.52.5.1128

[18] Hanifi-Moghaddam P, Kappler S, Seissler J, Muller-Scholze S, Martin S, Roep BO, et al Altered chemokine levels in individuals at risk of Type 1 diabetes mellitus. Diabet Med. 2006;23(2):156–63. 10.1111/j.1464-5491.2005.01743.x 16433713

[19] Ryden A, Stechova K, Durilova M, Faresjo M. Switch from a dominant Th1-associated immune profile during the pre-diabetic phase in favour of a temporary increase of a Th3-associated and inflammatory immune profile at the onset of type 1 diabetes. Diabetes Metab Res Rev. 2009;25(4):335–43. 10.1002/dmrr.958 19382103

[20] Stechova K, Bohmova K, Vrabelova Z, Sepa A, Stadlerova G, Zacharovova K, et al High T-helper-1 cytokines but low T-helper-3 cytokines, inflammatory cytokines and chemokines in children with high risk of developing type 1 diabetes. Diabetes Metab Res Rev. 2007;23(6):462–71. 10.1002/dmrr.718 17315139

[21] Alkanani AK, Rewers M, Dong F, Waugh K, Gottlieb PA, Zipris D. Dysregulated Toll-like receptor-induced interleukin-1beta and interleukin-6 responses in subjects at risk for the development of type 1 diabetes. Diabetes. 2012;61(10):2525–33. 10.2337/db12-0099 22751696 PMC3447890

[22] Nicoletti F, Conget I, Di Mauro M, Di Marco R, Mazzarino MC, Bendtzen K, et al Serum concentrations of the interferon-gamma-inducible chemokine IP-10/CXCL10 are augmented in both newly diagnosed Type I diabetes mellitus patients and subjects at risk of developing the disease. Diabetologia. 2002;45(8):1107–10. 10.1007/s00125-002-0879-5 12189440

[23] Roep BO, Kleijwegt FS, van Halteren AG, Bonato V, Boggi U, Vendrame F, et al Islet inflammation and CXCL10 in recent-onset type 1 diabetes. Clin Exp Immunol. 2010;159(3):338–43. 10.1111/j.1365-2249.2009.04087.x 20059481 PMC2819499

[24] Kawasaki E, Abiru N, Eguchi K. Prevention of type 1 diabetes: from the view point of beta cell damage. Diabetes Res Clin Pract. 2004;66 Suppl 1:S27–32. 15563975 10.1016/j.diabres.2003.09.015

[25] Wang C, Guan Y, Yang J. Cytokines in the Progression of Pancreatic beta-Cell Dysfunction. Int J Endocrinol. 2010;2010:515136 10.1155/2010/515136 21113299 PMC2989452

[26] Cieslak M, Wojtczak A, Cieslak M. Role of pro-inflammatory cytokines of pancreatic islets and prospects of elaboration of new methods for the diabetes treatment. Acta Biochim Pol. 2015;62(1):15–21. 25781159 10.18388/abp.2014_853

[27] Nakayama M, McDaniel K, Fitzgerald-Miller L, Kiekhaefer C, Snell-Bergeon JK, Davidson HW, et al Regulatory vs. inflammatory cytokine T-cell responses to mutated insulin peptides in healthy and type 1 diabetic subjects. Proc Natl Acad Sci U S A. 2015;112(14):4429–34. 10.1073/pnas.1502967112 25831495 PMC4394309

[28] Arif S, Tree TI, Astill TP, Tremble JM, Bishop AJ, Dayan CM, et al Autoreactive T cell responses show proinflammatory polarization in diabetes but a regulatory phenotype in health. J Clin Invest. 2004;113(3):451–63. 10.1172/JCI19585 14755342 PMC324541

[29] Steck AK, Johnson K, Barriga KJ, Miao D, Yu L, Hutton JC, et al Age of islet autoantibody appearance and mean levels of insulin, but not GAD or IA-2 autoantibodies, predict age of diagnosis of type 1 diabetes: diabetes autoimmunity study in the young. Diabetes Care. 2011;34(6):1397–9. 10.2337/dc10-2088 21562325 PMC3114355

[30] Steck AK, Dong F, Waugh K, Frohnert BI, Yu L, Norris JM, et al Predictors of slow progression to diabetes in children with multiple islet autoantibodies. J Autoimmun. 2016;72:113–7. 10.1016/j.jaut.2016.05.010 27255734 PMC4958563

[31] Hummel M, Bonifacio E, Schmid S, Walter M, Knopff A, Ziegler AG. Brief communication: early appearance of islet autoantibodies predicts childhood type 1 diabetes in offspring of diabetic parents. Ann Intern Med. 2004;140(11):882–6. 15172902 10.7326/0003-4819-140-11-200406010-00009

