# Supplementary figures and images for: Increased inflammation is associated with islet autoimmunity and type 1 diabetes in the Diabetes Autoimmunity Study in the Young (DAISY)
Source: PLoS One. 2017 Apr 5;12(4):e0174840. doi: 10.1371/journal.pone.0174840 (PMC5381877; doi:10.1371/journal.pone.0174840)

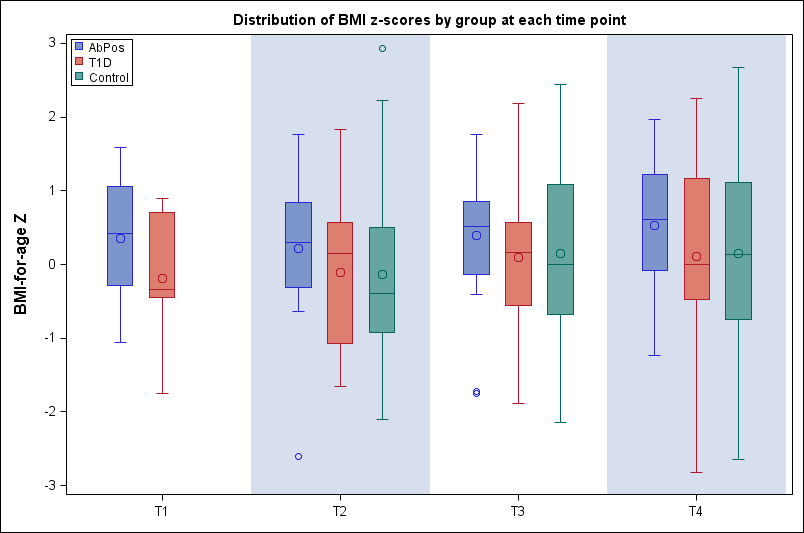

Supplement: S1 Fig — The BMI z-score distribution for each group at each time point. At time point 1 (T1) n = 11. Many participants are less than 1 year of age at T1 and length was not measured, therefore BMI and BMI z-score could not be calculated-no controls had recorded length measurements at T1. (TIF) [file pone.0174840.s001.tif]
