# Supplementary material for: Increased inflammation is associated with islet autoimmunity and type 1 diabetes in the Diabetes Autoimmunity Study in the Young (DAISY)
Source: PLoS One. 2017 Apr 5;12(4):e0174840. doi: 10.1371/journal.pone.0174840 (PMC5381877; doi:10.1371/journal.pone.0174840)
Supplement: S1 Table — Cytokine output in which greater that 50% of the data was censored was excluded from the analysis. (DOCX) [file pone.0174840.s002.docx]

**S1 Table: Percent censored and lower limit of detection (LLD) for each cytokine**

| **Cytokine** | **% Censored** | **LLD** | **Status** |
| --- | --- | --- | --- |
| IL-6 | 0.68 | 0.5 | Included |
| IP-10 | 0 | 3.2 | Included |
| MCP-1 | 0 | 1.8 | Included |
| IL-17 | 54.1 | 1.47 | Excluded |
| IL-1β | 74.3 | 0.16 | Excluded |
| IFN-α2a | 92.5 | 1.0 | Excluded |
| IL-1α | 74.3 | 0.2 | Excluded |
| IL-1ra | 96.2 | 8.0 | Excluded |
| IFN-γ | 33.2 | 0.1 | Included |
