# Supplementary material for: Increased inflammation is associated with islet autoimmunity and type 1 diabetes in the Diabetes Autoimmunity Study in the Young (DAISY)
Source: PLoS One. 2017 Apr 5;12(4):e0174840. doi: 10.1371/journal.pone.0174840 (PMC5381877; doi:10.1371/journal.pone.0174840)
Supplement: S2 Table — Univariate analysis of the log mean concentration for cytokines included in the analysis, by group and time point. *P-value of ≤ 0.05. (DOCX) [file pone.0174840.s003.docx]

**S2 Table: Log Mean Concentration of Cytokine by group and time**

**(unadjusted)**

|  | **Type 1 diabetes** | **IA positive** | **Control** | **p-value** |
| --- | --- | --- | --- | --- |
| **T1-Earliest available sample, prior to development of IA** | | | | |
|  | **N=22** | **N=25** | **N=25** |  |
| Log IL-6 | 0.99 ± 0.64 | 0.90 ± 0.69 | 1.1 ± 0.76 | 0.62 |
| Log IP-10 | 4.30 ± 0.48 | 4.36 ± 0.65 | 4.36 ± 0.64 | 0.91 |
| Log MCP-1 | 6.27 ± 0.36 | 6.24 ± 0.33 | 6.35 ± 0.28 | 0.47 |
| Log IFN-ү | -0.99 ± 0.97 | -0.73 ± 1.00 | -0.69 ±1.12 | 0.54 |
| **T2-Just prior to development of IA** | | | | |
|  | **N=19** | **N=25** | **N=25** |  |
| Log IL-6 | 1.05 ± 0.92 | 1.13 ± 0.81 | 1.02 ± 0.93 | 0.91 |
| Log IP-10 | 4.33 ± 0.63 | 4.41 ± 0.67 | 4.38 ± 0.62 | 0.91 |
| Log MCP-1 | 6.19 ± 0.32 | 6.01 ± 0.28 | 6.12 ± 0.37 | 0.17 |
| Log IFN-ү | -0.66 ± 1.19 | -0.95 ± 1.18 | -0.23 ± 1.28 | 0.12 |
| **T3-Just after development of IA** | | | | |
|  | **N=25** | **N=25** | **N=25** |  |
| Log IL-6 | 0.96 ± 0.75 | 1.04 ± 0.91 | 0.96 ± 0.70 | 0.92 |
| Log IP-10 | 4.30 ± 0.65 | 4.3 ± 0.46 | 4.18 ± 0.49 | 0.68 |
| Log MCP-1 | **6.13 ± 0.28** | **5.90 ± 0.25** | **6.05 ± 0.35** | **0.02*** |
| Log IFN-ү | -0.72 ± 1.52 | -1.62 ± 1.17 | -1.14 ± 1.07 | 0.0503 |
| **T4-Just prior to diagnosis of T1D, or most recent sample for IA/control groups** | | | | |
|  | **N=25** | **N=25** | **N=25** |  |
| Log IL-6 | 1.18 ± 0.52 | 0.88 ± 0.89 | 1.16 ± 0.73 | 0.28 |
| Log IP-10 | 4.41 ± 0.52 | 4.61 ± 0.79 | 4.26 ± 0.53 | 0.13 |
| Log MCP-1 | 6.07 ± 0.26 | 5.91 ± 0.47 | 6.03 ± 0.38 | 0.31 |
| Log IFN-ү | -0.98 ± 1.31 | -0.94 ± 1.79 | -1.46 ± 1.50 | 0.42 |
